# The Implementation of a Workplace Physical Exercise Program at a University

**DOI:** 10.3390/healthcare13172195

**Published:** 2025-09-02

**Authors:** Loreta Tobia, Maria Scatigna, Elio Tolli, Simona Delle Monache, Maria Giulia Vinciguerra, Leila Fabiani

**Affiliations:** 1 Department of Clinical Medicine, Public Health, Life Sciences and the Environment, University of L’Aquila, 67100 L’Aquila, Italy; loreta.tobia@univaq.it (L.T.); maria.scatigna@univaq.it (M.S.); leila.fabiani@univaq.it (L.F.); 2 Department of Biotechnological and Applied Clinical Sciences, University of L’Aquila, 67100 L’Aquila, Italy; simona.dellemonache@univaq.it (S.D.M.); mariagiulia.vinciguerra@univaq.it (M.G.V.)

**Keywords:** workplace health promotion, physical exercise, prevention, Non Communicable Diseases (NCDs) risk factors, work ability index, university workers

## Abstract

**Background**: Health promotion programs based on physical activity have gained increasing attention due to their potential to enhance employees’ physical and mental well-being, particularly in university settings, where academic and administrative staff are vulnerable to chronic stress, sedentary lifestyles, and work-related disorders. This study evaluates the effectiveness of the “University on the Move” program, an exercise-based workplace health promotion intervention implemented at the University of L’Aquila, Italy. **Methods**: An intervention study was conducted on 29 university employees participating in a supervised physical exercise program. Their anthropometric parameters, biochemical markers, cardiovascular risk factors, blood pressure, and work ability index were assessed at baseline (t0), three months (t1), and six months (t2), with a one-sample pre–post-test quasi-experimental design. **Results**: Significant improvements were observed in the cardiovascular risk factors, particularly in the female subgroup, e.g., the total cholesterol decreased by 20.8 mg/dL (*p* < 0.01), the LDL cholesterol decreased by 16.1 mg/dL (*p* < 0.01), and the fasting glucose decreased by 7.4 mg/dL (*p* < 0.01). Although the body mass index values remained stable, these metabolic improvements indicate beneficial effects independent of weight changes. The work ability index remained stable. The participation rates varied, with lower adherence to the training sessions. **Conclusions**: The study has some limitations (small sample size, no controlled design), all related to the primary aim of the preventive project targeted at the University employees who freely engaged in the protocol. Despite the low adherence (only about 30%), the metabolic improvements still suggest that structured workplace physical activity programs can positively impact employees’ health, even without significant weight loss, thus supporting the effectiveness of workplace health promotion and primary prevention interventions for an improved quality of life. Further research should explore long-term adherence and the organisational factors influencing participation.

## 1. Introduction

Health promotion is a multidimensional way of improving the physical, mental, and social well-being of individuals. It is fundamental for preventing diseases and for encouraging a healthy lifestyle. As highlighted by the Ottawa Charter of 1986, good health is a significant resource for social, economic, and personal development, and it is an essential aspect of quality of life [1]. In recent decades, a healthy lifestyle and physical activity have become global priorities in public health promotion. Growing scientific research has highlighted the importance of multidisciplinary and integrated disease prevention approaches, including behavioural, environmental, and policy interventions [2]. Medical and public health advancements have emphasised the need for evidence-based health policies and interventions [3]. This approach, known as evidence-based health promotion, allows for maximising the effectiveness of interventions, ultimately leading to reduced inequalities and optimised resource allocation. Numerous studies have shown that physical activity is one of the most effective forms of prevention against non-communicable chronic diseases, including cardiovascular diseases, respiratory diseases, type 2 diabetes, and obesity [4], which account for 50% of all pathologies. Physical activity also contributes to mental well-being, reduced stress levels, and an improved quality of life. These benefits are particularly relevant in workplace environments, making them ideal contexts to implement health promotion and disease prevention interventions, thus affecting workers’ physical, mental, and social well-being [5]. Such interventions can effectively control and prevent chronic diseases [6] and are associated with improved work productivity and reduced absenteeism [7]. This approach could also help workers adopt and spread these learned behaviours outside of the workplace [8]. In 1997, the World Health Organization launched the Workplace Health Promotion (WHP) program focused on targeting workplace organisational changes to encourage workers to adopt healthy lifestyles [9]. Additionally, the TWH^®^—Total Worker Health model, introduced by the American National Institute for Occupational Safety and Health (NIOSH), integrates protection from health and safety risks at work with efforts to prevent injuries and diseases to improve employee well-being [10].

The university setting represents a highly stressful workplace [11], with high workloads, academic and administrative responsibilities, and often significant pressure.

Indeed, recent studies have reported increased sitting time in university settings since COVID-19 [12]. Hence, there is a need for tailored WHP interventions.

Academic and administrative staff are particularly vulnerable to conditions such as chronic stress, sedentary lifestyles, and work-related disorders. Thus, they use compensatory coping strategies to deal with excessive demands [13].

This can negatively impact their quality of life and professional performance. Moreover, in many cases, they have limited access to health promotion initiatives in their workplace. Thus, promoting physical activity and a healthy lifestyle can become an effective strategy to improve employee mental health; to prevent chronic diseases and overweight; and to reduce the prevalence of hypertension, diabetes, and other leading risk factors [14]. Given the burden of cardiovascular disease, increasing the prevalence of healthy lifestyle choices is also a global imperative [15].

Thus, about 10 years ago the University of L’Aquila launched the ‘Ateneo in Salute’ (‘Healthy University’) initiative as part of the University Health Protection and Promotion program managed by the Occupational Safety and Security unit. The initiative aimed at improving physical, mental, and social well-being through surveillance and intervention activities, both at individual and group levels.

Unlike other worksite wellness interventions, such as Stanford’s BeWell program, our intervention is free and accessible to workers and students, and it is primarily focused on physical activity [16]. In 2020, the on-site ‘Ateneo in Movimento’ (University on the Move) project was launched. It consists of a weekly physical activity program intended for all employees and supported by a scientific evaluation protocol. This study aims to evaluate the potential impact of an exercise-based WHP intervention for improving physical health profiles and reducing the main risk factors of non-communicable diseases.

## 2. Materials and Methods

*Study design and intervention description:* An intervention study was carried out by administrating an exercise-based training program. The outcome evaluation consisted of a longitudinal single-sample design (one-group pre-test–post-test; quasi-experimental design), with a baseline measurement (t0, December 2023) before starting the program and two follow-ups at three months (t1, March 2024) and six months (t2, June 2024). The occupational health service offered the opportunity to participate to all male and female workers and students at the University of L’Aquila (Italy) aged between 18 and 70 years. The non-probabilistic sample consisted of 29 employees who voluntarily agreed to participate in the study. Therefore, the sample enrolment and the uncontrolled study design are due to the primary aim of the preventive project, i.e., the engagement of as many employees as possible in physical activity for their health benefits. This choice limited the outcome analysis, hindering the use of multivariate methods for separating the effects from other potential causes. After the fact, a power analysis showed that the study had 68% power to spot any LDL changes (α = 0.05; estimated effect size = −0.5 mg/dL).

The exercise program included 62 functional circuit training sessions. Training was twice weekly, comprising eight stations with a 1:1 work-to-rest ratio (30 s), repeated for three rounds, with a 10-min warm-up and a 10-min cool-down phase. The exercises were tailored to motor skills and physical capacity, including muscular strength and endurance, muscle flexibility, and cardiorespiratory fitness. Additionally, the program included 31 sessions of stretching and posture exercises, emphasising balance, mobility, proprioception, resistance, and respiratory dynamics.

The average participation rate for the “Circuit Training” subprogram was 13.9 sessions per participant (22.5% of the delivered sessions), whereas it was 9.7 sessions per participant (31.2%) for the “Stretching and Posture” subprogram.

*Anthropometric measurements and risk factors*: The weight and height were measured by using a bioelectrical impedance scale Wunder SA.BI.SRL, Model WBA300.

*Blood biochemical parameters*: Fasting venous blood samples were collected to determine the following parameters:

- Glucose: Measured in mg/dL using an enzymatic hexokinase method; the normal range is 65–110 mg/dL.
- Total Cholesterol: Measured in mg/dL using a colorimetric enzymatic method; the reference range is 0–200 mg/dL.
- HDL (High-Density Lipoprotein) and LDL (Low-Density Lipoprotein) Cholesterol: The HDL was determined via a direct analysis, while the LDL was calculated indirectly using the Friedewald formula. The normal ranges are 35–75 mg/dL for HDL and 0–130 mg/dL for LDL.
- Triglycerides: Measured in mg/dL using a colorimetric enzymatic method; the reference range is 60–130 mg/dL.
- C-Reactive Protein (CRP): Measured in mg/L using a high-sensitivity method (hs-CRP) to assess systemic inflammation and identify potential additional cardiovascular risks. The normal values are below 0.50 mg/dL.

*Cardiovascular risk*: The cardiovascular risk was calculated using the risk chart developed within the “The Heart Project—Progetto Cuore” of the Italian National Health Institute by estimating the percentage probability of a first major cardiovascular event (myocardial infarction or stroke) over the following 10 years. The chart accounts for eight risk factors: sex, age, diabetes, smoking habits, systolic blood pressure, total cholesterol, HDL cholesterol, and antihypertensive treatment [17]. The participants were categorised into six MCV (cardiovascular disease) risk categories based on their scores:

- MCV Risk I: <5%;
- MCV Risk II: 5–10%;
- MCV Risk III: 10–15%;
- MCV Risk IV: 15–20%;
- MCV Risk V: 20–30%;
- MCV Risk VI: >30%.

*Blood pressure values*: Blood pressure was measured using an automated, validated device subjected to regular maintenance and calibration. The measurements were interpreted according to the 2023 European Society of Hypertension guidelines [18].

*Work Ability Index (WAI)*: The WAI is an instrument developed by the Finnish Institute of Occupational Health, widely used in clinical occupational health and research to assess workability during health examinations and workplace surveys [19]. The index is determined through a questionnaire that considers a job’s demands and the worker’s health status and resources. The overall score ranges from 7 to 49. A subject’s work ability can then be classified as follows [20]:

- Poor: 7–27;
- Moderate: 28–36;
- Good: 37–43;
- Excellent: 44–49.

*Statistical analysis*: A statistical analysis was performed using STATA/BE 17.0 software. Frequencies were calculated for the categorical variables, and association tests were applied for the unmatched groups (chi-squared test, with Fisher’s correction) and matched groups (McNemar test, Cochrane Q test, symmetry test). Measures of central tendency and variability (arithmetic mean, standard deviation, and min-max range) were used for the quantitative variables.

Non-parametric tests were applied to evaluate the statistical significance (for unmatched data, Mann–Whitney test, Kruskal–Wallis test, and Cuzick’s trend test; for matched data, Wilcoxon test, Friedman test, and Skillings–Mack test) due to the absence of normal distribution tested with the Shapiro–Wilk test. The tests were two-tailed, with *p*-values below 0.05 considered statistically significant.

*Study participation and ethical considerations*: The study was approved by the Internal Review Board of the University of L’Aquila (approval no. 17/2020, dated 21 April 2020).

The participants were enrolled in the training program after obtaining a medical certificate of fitness for non-competitive sports from the university’s occupational health service. Paper and electronic informed consents for study participation and for processing personal data were obtained.

## 3. Results

### 3.1. Sample Description

At baseline, there were 29 participants, 24 females (82.8%) and 5 males (17.2%), with a median age of 54 years (range: 21–65 years). Slightly less than half of the participants were academics (48.3%), with the remainder comprising technical–administrative personnel (34.5%) and under- and post-graduate students (17.2%).

The most frequent risk conditions/diseases observed were dyslipidaemia (25.0% of women and 80.0% of men, *p* < 0.05), followed by hypertension (25.0% of women and 20.0% of men, not significant (n.s.)), thyroid disorders (29.2% of women and 0.0% of men, n.s.), cancer (8.3% of women and 0.0% of men, n.s.), diabetes (8.3% of women and 0.0% of men, n.s.), heart diseases (4.2% of women and 0.0% of men, n.s.), and osteoporosis (4.2% of women and 0.0% of men, n.s.).

More than half of the women were in the menopausal stage (52.2%) (Table 1).

Approximately 10% of the total sample smoked tobacco (12.5% of the female and none of the male subsamples), with an average of 3.3 cigarettes smoked per day (range: 1–5 cigarettes per day). Meanwhile, 27.6% of the sample had quit this habit (60.0% of the men and 20.8% of the women). Occasional alcohol consumption was reported by 41.7% of the women and 100.0% of the men (*p* < 0.05). Overall, 93.1% of the total sample engaged in some form of regular physical activity (sport, exercise, or recreational activity), with slightly higher levels in the men (100.0% vs. 91.7% of the women, n.s.), and also in terms of the weekly frequency (3.2 vs. 2.9 times per week, respectively, n.s.) and total minutes per week (228.0 vs. 188.2 min per week, respectively, n.s.) (Table 2).

Overall, 54.2% of the women and 100.0% of the men underwent drug therapy (difference not significant, *p* = 0.0548), with an average of 0.8 and 1 medication per person, respectively (n.s.). The most frequently used medications were antihypertensives (five cases), hypolipidemic agents (four cases), and, less frequently, anti-inflammatories (two), antidiabetic drugs (two), hormones (two), aspirin cardio (two), along with other medications used by individuals (e.g., for autoimmune disease treatment, antacids, etc.). Among the respondents, 66.7% of the women and 40.0% of the men reported taking dietary supplements (difference not significant), including preparations containing minerals (e.g., Mg and K) in eight cases, Vitamin D in seven cases, B-complex vitamins in six cases, antioxidants (Vitamins C, A, and E) in five cases, iron in three cases, and others (e.g., probiotics, immunostimulants) in two cases (Table 2).

### 3.2. Anthropometric, Biochemical, and Physiological Variables

At baseline, both in the female and male subgroups, the average BMI (body mass index) values fell within the normal weight range and remained almost constant over time. Among the women, it was equal to 24.4 ± 4.4 Kg/m^2^ at t0, 24.3 ± 4.3 Kg/m^2^ at t1, and 24.4 ± 4.2 Kg/m^2^ at t2; among the men, it was 25.3 ± 3.6 Kg/m^2^, 25.3 ± 3.0 Kg/m^2^, and 25.1 ± 3.3 Kg/m^2^, respectively. Overall, the proportion of overweight subjects was higher among the men: 60.0% of the men were overweight at all three observation points, compared to 29.2% at t0 and t1 and 30.4% at t2 among the women. None of the men were obese, whereas three of the female subjects were obese at t0, t1, and t2. No significant differences were found among the temporal trends, either in the Skillings–Mack test for BMI or in the Cochran Q test for weight categories in either the women or men (Table 3).

At baseline, 50.0% of the women and 20.0% of the men had plasma concentrations of total cholesterol and LDL cholesterol higher than the normal limits for the adult population. These proportions decreased in both subgroups over time. Specifically, in the female subgroup, for total cholesterol, the proportions decreased to 47.8% (t1) and 30.4% (t2) in a statistically non-significant manner (*p* = 0.0907 for the Cochran Q test, with a greater effect in the partial comparison of t0 vs. t2, *p* = 0.0588), and for LDL cholesterol, to 47.8% (t1) and 21.7% (t2) in a statistically significant manner (*p* < 0.01 for the Cochran Q test, with statistical significance for the t0 vs. t2 comparison, *p* < 0.01; and t1 vs. t2, *p* < 0.05). Only 4.2% of the women and none of the men showed HDL cholesterol levels outside the normal range (either high or low) at baseline, and this proportion only increased among the women, rising to 8.2% at both t1 and t2 (n.s. for the Cochran Q test). The plasma concentration of triglycerides was above the normal range in 8.3% of the women and 20.0% of the men at baseline. In both subgroups, the differences in the percentages observed over time were not statistically significant. For the women they were 13.0% at t1 and 4.4% at t2; among the men, they were 0.0% and 40.0%, respectively. At baseline, 8.3% of the women had fasting blood glucose values above the threshold for normality; this proportion remained the same at t1 and decreased to 0.0% at t2 (n.s.); none of the men had abnormal glucose values at baseline or at subsequent times. The CRP levels were higher than normal in 8.3% of the women and 20.0% of the men at baseline, and while they normalised in all the men, among the women, the proportion with values above the normal threshold increased (9.1% at t1 and 17.4% at t2). These differences were not statistically significant for the Cochran Q test in either subgroup (Table 4).

On average, the quantitative values of the haematochemical parameters showed a reduction from baseline to t2 in both the women and men, except for the triglycerides and CRP (the latter only in the female subgroup). In the women, the average blood concentration of total cholesterol changed from 199.5 ± 41.1 mg/dL (at t0) to 199.1 ± 41.0 mg/dL (at t1) to 178.7 ± 34.9 mg/dL (at t2) in a statistically significant manner (*p* < 0.01 for the Skillings–Mack test), while in the men it changed from 183.2 ± 60.5 mg/dL to 166.4 ± 22.9 mg/dL to 161.4 ± 23.9 mg/dL in a non-significant manner. In the women, the HDL cholesterol changed from 59.5 ± 10.2 mg/dL (at t0) to 61.1 ± 11.0 mg/dL (at t1) to 55.3 ± 10.4 mg/dL (at t2) in a statistically significant manner (*p* < 0.001), while in the men the HDL cholesterol changed from 50.6 ± 12.2 mg/dL to 53.6 ± 11.5 mg/dL to 47.4 ± 7.0 mg/dL in a non-significant manner. In the women, the LDL cholesterol varied from 120.5 ± 38.7 mg/dL (at t0) to 124.1 ± 36.8 mg/dL (at t1) to 104.4 ± 33.6 mg/dL (at t2) in a statistically significant manner (*p* < 0.01), while in the men the LDL cholesterol changed from 109.0 ± 55.5 mg/dL to 96.4 ± 18.5 mg/dL to 91.2 ± 23.0 mg/dL in a non-significant manner. In the women, the triglycerides changed from 78.1 ± 26.4 mg/dL (at t0) to 93.0 ± 36.8 mg/dL (at t1) to 84.9 ± 37.3 mg/dL (at t2) in a statistically significant manner (*p* < 0.05), while in the men the triglycerides changed from 85.2 ± 37.3 mg/dL to 84.4 ± 19.0 mg/dL to 107.0 ± 45.9 mg/dL in a non-significant manner. In the women, the fasting glucose changed from 90.6 ± 18.0 mg/dL (at t0) to 86.1 ± 18.7 mg/dL (at t1) to 83.2 ± 10.5 mg/dL (at t2) in a statistically significant manner (*p* < 0.01), while in the men it changed from 90.0 ± 14.4 mg/dL to 81.6 ± 10.9 mg/dL to 80.8 ± 14.5 mg/dL in a non-significant manner. In the women the C-reactive protein changed from 0.27 ± 0.58 mg/100 mL (at t0) to 0.20 ± 0.28 mg/100 mL (at t1) to 0.30 ± 0.42 mg/100 mL (at t2), while in the men it changed from 0.32 ± 0.59 mg/100 mL to 0.04 ± 0.04 mg/100 mL to 0.13 ± 0.07 mg/100 mL, in both subgroups in a non-significant manner (Table 5).

Table 6 shows the distribution of the entire sample (normotensive subjects and hypertensive subjects on pharmacological therapy, who did not undergo any changes in therapy during the study) across risk categories based on the blood pressure values measured during the baseline assessments and subsequent follow-ups, as well as the Heart Score calculated by using the haematochemical and blood pressure parameters, according to the international guidelines of the Italian National Institute of Health [17].

At baseline, 70.8% of the women and 60.0% of the men exhibited ‘non-normal’ blood pressure values (altered or elevated to varying degrees). These proportions changed during the follow-ups, amounting to 54.2% and 60.0% (t1) and 30.4% and 40.0% (t2), respectively, with statistically significant differences observed in the women’s subgroup (*p* < 0.01 for the Cochrane Q test). Similarly, at baseline, 11.1% of the women and 40.0% of the men had a Heart Score equal to or greater than 5.0 (the lowest risk level). These proportions were 11.1% and 20.0% (t1) and 5.9% and 40.0% (t2) at the follow-ups, thus showing discordant trends and no statistically significant changes in either sex subgroup.

The quantitative values of blood pressure decreased in both sexes over time: for the women, the systolic blood pressure varied from 118.1 ± 8.6 mmHg (t0) to 113.8 ± 9.5 mmHg (t1) to 110.7 ± 10.4 mmHg (t2), and the diastolic blood pressure from 75.0 ± 8.2 mmHg (t0) to 68.3 ± 9.0 mmHg (t1) to 70.0 ± 7.4 mmHg (t2), with both parameters showing statistically significant reductions (*p* < 0.01, Skillings–Mack test); for the men, the systolic blood pressure varied from 125.0 ± 15.0 mmHg (t0) to 114.0 ± 5.5 mmHg (t1) to 116.0 ± 13.4 mmHg (t2), and the diastolic blood pressure from 76.0 ± 8.9 mmHg (t0) to 70.0 ± 7.1 mmHg (t1) to 71.0 ± 7.4 mmHg (t2), with both parameters decreasing but not in a statistically significant manner.

Similarly, the overall Heart Score values decreased: for the women, the values ranged from 2.2 ± 1.7% (t0) to 2.0 ± 1.8% (t1) to 1.7 ± 1.4% (t2), with a statistically significant reduction (*p* < 0.01, Skillings–Mack test), while for the men the values ranged from 4.6 ± 2.3% (t0) to 3.5 ± 1.9% (t1) to 3.9 ± 2.3% (t2), but the reduction was not statistically significant (Table 7).

### 3.3. Work Ability Index

The average score on the WAI was categorised as ‘good’ for the women at baseline and at all three observation points, with scores of 41.1 ± 5.0 (t0), 40.4 ± 5.1 (t1), and 39.9 ± 6.6 (t2). For the men, the score was categorised as ‘excellent,’ with scores of 44.2 ± 4.4 (t0), 46.0 ± 2.9 (t1), and 44.6 ± 3.5 (t2). In both subgroups, the differences observed were not statistically significant according to the Skillings–Mack test (Table 8).

## 4. Discussion

In recent times, health promotion in workplaces has gained paramount importance. In 2020, to counteract the physical inactivity linked to COVID-19, the International Society for Physical Activity and Health included workplaces among the eight investment areas that work for physical activity, supported by strong evidence of effectiveness and applicable at a global level [21]. The findings obtained from a systematic review by Zhang et al. in 2025 demonstrate that workplace interventions exhibit varying degrees of effectiveness for improving employee health status, optimising body composition, enhancing physical function, promoting mental well-being, and improving work-related outcomes [22].

Academic environments appear to have received particular focus, as evidenced by the numerous initiatives launched worldwide, including our “University on the Move” project by the University of L’Aquila. Many universities, especially in North America and Europe, have invested in building gyms, fitness centres, and spaces dedicated to physical activity. For example, Stanford University has implemented the “BeWell” program, which includes access to gyms, fitness classes, and walking paths on campus [16]. Some universities have introduced flexible work policies to facilitate employee participation in physical activities during work hours. A successful example is the University of Queensland in Australia, which introduced scheduled breaks for physical activity as part of the “Active Workplaces” project that can include short yoga sessions, stretching, or group walks [23]. Universities in Northern Europe, particularly in countries like Sweden and Norway, are at the forefront of health promotion through awareness campaigns. These programs, often funded by the universities themselves or by local governments, combine nutrition and mental health education with financial incentives for participation in fitness programs [24]. The Karolinska Institute in Sweden has developed an initiative to encourage employees to be healthier in mind and body, providing a wellness allowance that staff can use for a wide range of exercise-based activities or other forms of health promotion, such as active transportation, and which was increased starting in January 2024 [25]. In the last few years, awareness has grown that health promotion should not be limited to physical activity but should embrace a holistic approach. In this context, many universities in Asia, such as the National University of Singapore, have launched holistic wellness programs, like the one called “(kind)mind”, aimed to destigmatise mental health conditions and combine physical activity with mindfulness initiatives, mindful eating, and psychological support [26].

In Italy, several universities have also begun to implement programs to promote physical activity and healthy lifestyles. Health promotion in workplaces is also included among the Predefined Programs 3 (PP3) of the National Prevention Plan 2020–2025. In fact, among other objectives, it aims to encourage the conscious adoption of a healthy and active lifestyle at all ages and across various life and work settings by integrating individual change with social transformation [27]. In February 2025, the Italian Ministry of Health published the “Policy document on health promotion in workplaces of the public administration”, with the goal of enhancing the transferability and replicability of intervention models [28]. The most common interventions involve the creation of sports infrastructure, the organisation of awareness-raising campaigns, and the promotion of workplace wellness policies. Health values are often studied in connection with preventive habits. Thus, the University of L’Aquila has investigated how these factors relate to the behaviours and attitudes of its workers, particularly in relation to SARS-CoV-2 infection and the manifestation of symptoms [29]. However, these initiatives are not yet uniformly distributed across the country, and there is still room for improvement in terms of both distribution and participation. Indeed, information on the potential benefits of health promotion in the workplace, aligned with the perceptions and needs of occupational physicians, seems necessary to successfully implement health promotion interventions [30]. Many Italian universities have invested in creating sports facilities accessible to both employees and students, by offering gyms on their campuses, promoting access to affiliated sports facilities, and by integrating activities such as swimming, fitness, and soccer. These initiatives aim to encourage an active lifestyle to counteract the sedentary aspects of academia. The University of Bologna has launched the “UNIFIT” program to implement active workplace breaks and interrupt sedentary behaviour among workers during working hours [31]. A further example is the University of Florence that, together with 10 European universities, has become part of The European University for Well-Being (EUniWell), an alliance established by the European Commission with a mission to understand, enhance, measure, and rebalance well-being at individual, community, and societal levels. Their activities involve research arenas and work packages that engage academics, administrative staff, and students from all partner universities on three different matters: health and well-being; individual and social well-being; and environment, urbanity, and well-being [32].

Despite progress, universities face several challenges in implementing health promotion programs. Among the main barriers are employees’ low participation, primarily due to high workloads and a lack of time for physical activity [12,33] and cultural resistance, which challenges the widespread adoption of such programs [34]. Poor adherence was also a critical factor in our intervention, and it most likely influenced the effects on the different outcome variables. This could be explained by the newness of the experience for the employees involved during the first year of the activation of the program and their need to reach a balance within their routine. Possible solutions to increase adherence are educational messages to enhance awareness of health benefits, sharing data on the measured results, and negotiating greater flexibility for exercise session scheduling and support from university administrators [33].

Considering the clinical data, the present study’s sample was in good physical health and originated from a non-clinical and/or non-particularly unfavourable physical health setting, a university workplace. Specifically, the women exhibited a higher number and variety of pathologies, and slightly more than half were in menopause, as confirmed by the Global Burden of Disease Study 2021, which showed how women are more susceptible to chronic diseases than men [35].

Cigarette smoking was uncommon, observed only among the women, with a maximum daily consumption of no more than five cigarettes/day, in contrast with the national averages from the Italian data for the 2022–2023 biennium, which showed that smoking habits were more prevalent among men (28.3%; 95% CI: 27.7–28.9%) than women (20.7%; 95% CI: 20.1–21.3%) [36].

Alcohol consumption was more frequent among the men, as confirmed by data from the Italian surveillance system PASSI (Progress of Local Health Authorities for Health in Italy), on which men had the highest percentage of both binge drinking and high-risk alcohol consumption [36]; however, in our sample, the questions did not assess quantities or problematic use/abuse.

On average, the sample fell within a normal weight range, although the mean BMI value was close to the upper limit of this category (i.e., 25.0). No changes were observed across the three time points in either the BMI values or the distribution across weight categories (normal weight, overweight, and obesity), indicating no detectable weight loss. These findings align with the average Italian data reported by the PASSI surveillance system promoted by the Italian National Institute of Health. National-level temporal analyses conducted during 2022–2023 did not show significant variations in overweight and obesity prevalence. Based on their analysis, 40.9% of men are overweight, and 11.1 are obese. Similarly, among women, 24.5% are overweight, and 9.7% are obese [36].

Statistically significant improvements over time were observed in the haematochemical and blood pressure parameters, particularly in the larger female subsample. These findings refer to a sample that also included cases of hypertension and dyslipidaemia under pharmacological treatment, yet improvements were still observed, and they are consistent with other health promotion interventions implemented in various settings, such as walking groups, which, according to a study by Hanson et al. in 2015, were shown to be safe and effective. Among other benefits, a reduction in the total cholesterol of −0.11 mmol/L was reported (−0.22 to −0.01) [37].

An abnormal trend was observed in the HDL cholesterol levels, which showed a decrease over time. This finding may be related to the type of physical activity performed. The participants in our study mainly followed a resistance exercise program, which is associated with a smaller improvement in HDL cholesterol levels compared to aerobic activity [38].

Interestingly, as shown above, the improvements appear to be independent of the unchanged weight status. Thus, although the level of energy expenditure on physical exercise did not lead to a change in weight, there were still some health benefits. In 2017, after a short high-intensity interval training program, Ouerghi et al. reported a significant reduction in body mass (−1.62%) and in fat mass (−1.59%) in the obese but not in the normal-weight subjects [39]. On the other hand, a 2015 study by Otha et al. did not reveal any significant variation in BMI, but did show a significant improvement in glucose levels after a bench step exercise program [40].

The WAI index showed a consistent and average placement across the three time points: the women were in the ‘good’ category and the men were in the ‘excellent’ category, so further improvement was unexpected due to a potential ceiling effect. The high-baseline WAI scores (‘good’ to ‘excellent’) capped the ability to see improvements, aligning with studies on knowledge workers [41].

Studies in both university and non-university settings have shown that the WAI tends to be higher in occupations with greater mental than physical demands. Additionally, some studies have correlated the WAI with physical activity, highlighting how the WAI remains high and stable in those who participate in physical activity programs [41,42]. Unlike the study conducted by Da Silva et al. in 2024 on a university campus in Southern Brazil, where the professors presented with more favourable sociodemographic and lifestyle aspects and higher workability compared to the outsourced workers [43], the differences were not significant in the WAI scores observed in our study for sociodemographic and job position variables (higher scores in men, younger individuals, and teaching staff compared to technical–administrative staff). However, the influence of the small sample size cannot be excluded.

### Limitations

The study has notable limitations. It was conducted with a pre–post design on a single sample (non-controlled and non-randomised). The health goals for all the workers involved in the research were considered the priority, and we decided to adopt a quasi-experimental evaluative approach involving all the subjects enrolled in the exercise program. The small sample size reduced the statistical power. Specifically, some improvement trends were detectable in the men and in the women, but not at a statistically significant level (e.g., haematochemical and blood pressure parameters). Furthermore, due to the small sample size, it was not possible to apply multivariate statistical analysis models (e.g., multiple regression models), which would have allowed us to control for confounding factors. Moreover, the dose–response effect could be considered a critical aspect of our study due to the poor participation rate in the exercise-based training program (on average, below or near 30% of the weekly sessions).

The use of paired data tests allowed for the evaluation of the improvement in the entire sample, considering the individual starting points, which may have varied depending on their clinical status. However, a comparison of benefits between the groups of individuals with health conditions (i.e., hypertension, hyperglycaemia, and dyslipidaemia) and healthy individuals was not possible due to the limited size of the subgroups. The use of medication(s)/dietary supplements were documented, but they lacked any analytic control because of sample size problems. The sample also displayed qualitative heterogeneity because it was predominantly female with a wide age range: the median age was above 50 years, and while both younger and older age groups were represented, the small subsample sizes prevented the examination of the impact of age on the different associations. However, similar studies have also had a small sample size, such as those conducted by Ohta et al. in 2014 and 2015 [20,40].

Despite the small sample size and the caution needed when low-power tests are used, as in our analysis with a non-parametric test, initial meaningful results have been obtained, which is encouraging for further intervention and research investigations.

## 5. Conclusions

Our study examines one of the few examples that offer spaces and programs for physical activity in the workplace supported by internal resources. Although the study sample did not present with unfavourable clinical conditions, meaningful positive effects on their physical health were observed, with improvements in their physiological and haematochemical markers, thus encouraging the implementation of this kind of intervention in primary prevention and health promotion. However, the methodological limitations of the evaluation study, such as the small and inhomogeneous sample (especially the sex imbalance) and uncontrolled design, reduce the external validity of the results. Refining the research question to fill this gap by means of a randomised controlled design and use of multivariate analyses might confirm the benefits of an exercise program in university workplaces and evaluate its impacts on the different categories of employees in this setting (i.e., teaching and research personnel, administrative staff).

Future perspectives should focus on the attitudinal correlates of a structured workplace intervention, the organisational factors (e.g., the incorporation of movement at other times of the day), changes in other lifestyle behaviours, and the impact on the workers’ communities. Studying these correlates could lead to improving offerings, expanding participation, ensuring sustainability, and facilitating the transfer of good practices.

## Figures and Tables

**Table 1:** General and anamnestic characteristics of the sample.

		Women (24)	Men (5)	Total (29)	Sign.
Age (years)	Median (range)	53 (21–65)	60 (47–61)	54 (21–65)	n.s. ^(a)^
	≤45 years 46–59 years ≥60 years	6 (25.0%) 11 (45.8%) 7 (29.2%)	0 (0.0%) 2 (20.0%) 3 (60.0%)	6 (20.7%) 13 (44.8%) 10 (34.5%)	n.s. ^(b)^
Occupation	Student/PhD Student Researcher/Professor Technician/Clerk	5 (20.8%) 10 (41.7%) 9 (37.5%)	0 (0.0%) 4 (80.0%) 1 (20.0%)	5 (17.2%) 14 (48.3%) 10 (34.5%)	n.s. ^(b)^ n.s. ^(b)^ n.s. ^(b)^
Family History	Diabetes Tumours CV diseases	10 (41.7%) 18 (75.0%) 12 (52.2%)	4 (80.0%) 1 (20.0%) 1 (20.0%)	14 (48.3%) 19 (65.5%) 13 (46.4%)	n.s. ^(b)^ *p* < 0.05 ^(b)^ n.s. ^(b)^
Medical History/Risk Factors	Dyslipidaemia Hypertension Thyroid diseases Cancer Diabetes Heart diseases Osteoporosis Menopause	6 (25.0%) 6 (25.0%) 7 (29.2%) 2 (8.3%) 2 (8.3%) 1 (4.2%) 1 (4.2%) 12 (52.2%)	4 (80.0%) 1 (20.0%) 0 (0.0%) 0 (0.0%) 0 (0.0%) 0 (0.0%) 0 (0.0%) -	10 (34.5%) 7 (24.1%) 7 (24.1%) 2 (6.9%) 2 (6.9%) 1 (3.5%) 1 (3.5%) -	*p* < 0.05 ^(b)^ n.s. ^(b)^ n.s. ^(b)^ n.s. ^(b)^ n.s. ^(b)^ n.s. ^(b)^ n.s. ^(b)^

^(a)^ Non-parametric test of rank sums (Mann–Whitney); ^(b)^ chi-square test with Fisher’s correction.

**Table 2:** Lifestyle, pharmacological therapies, and use of dietary supplements in the sample.

		Women (24)	Men (5)	Total (29)	Sign.
Tobacco Smoking	Yes Ex No	3 (12.5%) 5 (20.8%) 2 (40.0%)	0 (0.0%) 3 (60.0%) 2 (40.0%)	3 (10.3%) 8 (27.6%) 18 (62.1%)	n.s. ^(a)^ n.s. ^(a)^ n.s. ^(a)^
No. of Cigarettes/Day	(mean, range)	3.3 (1–5)	-	3.3 (1–5)	n.s. ^(a)^
Alcohol Consumption	(Occasional)	10 (41.7%)	5 (100.0%)	15 (51.7%)	*p* < 0.05 ^(a)^
Regular Physical Activity No. of Times/Week Total Minutes/Week	Mean (range) Mean (range)	22 (91.7%) 2.9 (1–7) 188.2 (60–420)	5 (100.0%) 3.2 (2–4) 228.0 (180–360)	27 (93.1%) 2.9 (1–7) 195 (60–420)	n.s. ^(a)^ n.s. ^(b)^ n.s. ^(b)^
Pharmacological Therapies No. of Drugs	Mean (range)	13 (54.2%) 0.8 (0-5)	5 (100.0%) 1 (1-1)	17 (58.6%) 0.9 (0-5)	n.s. ^(a)^ n.s. ^(b)^
Use of Supplements		16 (66.7%)	2 (40.0%)	18 (62.1%)	n.s. ^(a)^

^(a)^ Chi-square test with Fisher’s correction; ^(b)^ non-parametric Mann–Whitney rank-sum test. Ex former smoker.

**Table 3:** Trends over time in anthropometric measurements for estimating body mass, stratified by sex. Arithmetic means ± standard deviation and distribution of participants by weight risk categories.

	Women (24)				Men (5)			
	t0	t1	t2	Sign.	t0	t1	t2	Sign.
No.	24	24	23		5	5	5	
BMI (Kg/m^2^)	24.4 ± 4.4	24.3 ± 4.3	24.4 ± 4.2	n.s. ^(a)^	25.3 ± 3.6	25.3 ± 3.0	25.1 ± 3.3	n.s. ^(a)^
Normal Weight (%)	14 (58.3%)	14 (58.3%)	13 (56.5%)	n.s. ^(b)^	2 (40.0%)	2 (40.0%)	2 (40.0%)	n.s. ^(b)^
Overweight (%)	7 (29.2%)	7 (29.2%)	7 (30.4%)	n.s. ^(b)^	3 (60.0%)	3 (60.0%)	3 (60.0%)	n.s. ^(b)^
Obesity (%)	3 (12.5%)	3 (12.5%)	3 (13.0%)	n.s. ^(b)^	0 (0.0%)	0 (0.0%)	0 (0.0%)	n.s. ^(b)^

^(a)^ Skillings–Mack test; ^(b)^ Cochran Q test.

**Table 4:** Proportion of subjects in categories defined by normality cut-offs of haematochemical parameters, stratified by sex and observation time, and significance of differences on paired Cochran Q test.

	Women (24)				Men (5)			
	t0 (24)	t1 (23)	t2 (23)	Sign.	t0 (5)	t1 (5)	t2 (5)	Sign.
Chol TOT ≥200 mg/dL	12 (50.0%)	11 (47.8%)	7 (30.4%)	*p* = 0.091	1 (20.0%)	0 (0.0%)	0 (0.0%)	N/A
Chol HDL <35 or >75 mg/dL	1 (4.2%)	2 (8.7%)	2 (8.7%)	n.s.	0 (0.0%)	0 (0.0%)	0 (0.0%)	N/A
Chol LDL ≥130 mg/dL	12 (50.0%)	11 (47.8%)	5 (21.7%)	*p* < 0.01	1 (20.0%)	0 (0.0%)	0 (0.0%)	N/A
Triglycerides ≥130 mg/dL	2 (8.3%)	3 (13.0%)	1 (4.4%)	n.s.	1 (20.0%)	0 (0.0%)	2 (40.0%)	n.s.
Glucose ≥110 mg/dL	2 (8.3%)	2 (8.7%)	0 (0.0%)	n.s.	0 (0.0%)	0 (0.0%)	0 (0.0%)	N/A
CRP ≥0.5 mg/100 mL	2 (8.3%)	2 (9.1%) ^§^	4 (17.4%)	n.s.	1 (20.0%)	0 (0.0%)	0 (0.0%)	N/A

^§^ There were 22 samples for CRP; N/A = not applicable.

**Table 5:** Trends over time of haematochemical parameters stratified by sex. Arithmetic means ± standard deviation and significance of differences in non-parametric Skillings–Mack test for paired data.

	Women (24)				Men (5)			
	t0 (24)	t1 (23)	t2 (23)	Sign.	t0 (5)	t1 (5)	t2 (5)	Sign.
Chol TOT (mg/dL)	199.5 ± 41.1	199.1 ± 41.0	178.7 ± 34.9	*p* < 0.01	183.2 ± 60.5	166.4 ± 22.9	161.4 ± 23.9	n.s.
Chol HDL (mg/dL)	59.5 ± 10.2	61.1 ± 11.0	55.3 ± 10.4	*p* < 0.001	50.6 ± 12.2	53.6 ± 11.5	47.4 ± 7.0	n.s.
Chol LDL (mg/dL)	120.5 ± 38.7	124.1 ± 36.8	104.4 ± 33.6	*p* < 0.01	109.0 ± 55.5	96.4 ± 18.5	91.2 ± 23.0	n.s.
Triglycerides (mg/dL)	78.1 ± 26.4	93.0 ± 36.8	84.9 ± 37.3	*p* < 0.05	85.2 ± 37.3	84.4 ± 19.0	107.0 ± 45.9	n.s.
Glucose (mg/dL)	90.6 ± 18.0	86.1 ± 18.7	83.2 ± 10.5	*p* < 0.01	90.0 ± 14.4	81.6 ± 10.9	80.8 ± 14.5	n.s.
CRP (mg/100 mL)	0.27 ± 0.58	0.20 ± 0.28 ^§^	0.30 ± 0.42	n.s.	0.32 ± 0.59	0.04 ± 0.04	0.13 ± 0.07	n.s.

^§^ There were observations.

**Table 6:** Proportion of subjects in different blood pressure and Heart Score categories [17], stratified by sex, observation time, and significance of differences using non-parametric Cochran Q test for paired data.

	Women (24)				Men (5)			
	t0 (24)	t1 (23)	t2 (23)	Sign.	t0 (5)	t1 (5)	t2 (5)	Sign.
**Blood Pressure** Normal Non-Normal	7 (29.2%) 17 (70.8%)	11 (45.8%) 13 (54.2%)	16 (69.6%) 7 (30.4%)	*p* < 0.01	2 (40.0%) 3 (60.0%)	2 (40.0%) 3 (60.0%)	3 (60.0%) 2 (40.0%)	n.s.
Heart Score * I (<5%) Beyond I (≥5%)	16 (88.9%) 2 (11.1%)	15 (88.2%) 2 (11.1%)	16 (94.1%) 1 (5.9%)	n.s.	3 (60.0%) 2 (40.0%)	4 (80.0%) 1 (20.0%)	3 (60.0%) 2 (40.0%)	n.s.

* For the Heart Score at t0, only 18 units were observed, and at times t1 and t2, only 17 units were observed.

**Table 7:** Trends over time in systolic and diastolic blood pressure values and Heart Score stratified by sex. Arithmetic means ± standard deviation and significance of differences on non-parametric Skillings–Mack test for repeated measures.

	Women (24)				Men (5)			
	t0 (24)	t1 (23)	t2 (23)	Sign.	t0 (5)	t1 (5)	t2 (5)	Sign.
Systolic (mmHg)	118.1 ± 8.6	113.8 ± 9.5	110.7 ± 10.4	*p* < 0.01	125.0 ± 15.0	114.0 ± 5.5	116.0 ± 13.4	n.s.
Diastolic (mmHg)	75.0 ± 8.2	68.3 ± 9	70.0 ± 7.4	*p* < 0.01	76.0 ± 8.9	70.0 ± 7.1	71.0 ± 7.4	n.s.
Heart Score (%)	2.2 ± 1.7 ^a^	2.0 ± 1.8 ^b^	1.7 ± 1.4 ^b^	*p* < 0.01	4.6 ± 2.3	3.5 ± 1.9	3.9 ± 2.3	n.s.

^a^ only 18 units observed; ^b^ only 17 units observed.

**Table 8:** Trends over time for the work ability index over the observation periods, stratified by sex. Arithmetic means ± standard deviation of the scores and distribution of the participants within the ability categories [18].

	Women (24)				Men (5)			
	t0 (24)	t1 (23)	t2 (23)	Sign.	t0 (5)	t1 (5)	t2 (5)	Sign.
**WAI Score** (range 7–49)	41.1 ± 5.0	40.4 ± 5.1	39.9 ± 6.6	n.s. ^(a)^	44.2 ± 4.4	46.0 ± 2.9	44.6 ± 3.5	n.s. ^(a)^
**WAI Categories** 1—Poor 2—Moderate 3—Good 4—Excellent	1 (4.2%) 0 (0.0%) 14 (58.3%) 9 (37.5%)	1 (4.4%) 2 (8.7%) 15 (65.2%) 5 (21.7%)	1 (5.9%) 2 (11.8%) 10 (58.8%) 4 (23.5%)	n.s. ^(b)^	0 (0.0%) 0 (0.0%) 1 (20.0%) 4 (80.0%)	0 (0.0%) 0 (0.0%) 1 (20.0%) 4 (80.0%)	0 (0.0%) 0 (0.0%) 2 (40.0%) 3 (60.0%)	n.s. ^(b)^

^(a)^ Skillings–Mack test; ^(b)^ symmetry test for comparison of WAI-t0 vs. WAI-t1, WAI-t0 vs. WAI-t2, and WAI-t1 vs. WAI-t2.

## Data Availability

The datasets generated and analysed during the current study are not publicly available due to restrictions associated with anonymity of participants but are available from the corresponding author on reasonable request.

## Abbreviations

The following abbreviations are used in this manuscript:

NCDs	Non Communicable Diseases
WHP	Workplace Health Promotion
TWH	Total Worker Health
NIOSH	National Institute for Occupational Safety and Health
HDL	High-Density Lipoprotein
LDL	Low-Density Lipoprotein
CRP	C-Reactive Protein
MCV	Cardiovascular Disease
WAI	Work Ability Index
n.s.	Not Significant
BMI	Body Mass Index
PP3	Predefined Program 3
EUniWell	The European University for Well-Being
PASSI	Progress of Local Health Authorities for Health in Italy
Sign.	Significance
